# Notch Signaling Affects Oral Neoplasm Cell Differentiation and Acquisition of Tumor-Specific Characteristics

**DOI:** 10.3390/ijms20081973

**Published:** 2019-04-22

**Authors:** Keisuke Nakano, Kiyofumi Takabatake, Hotaka Kawai, Saori Yoshida, Hatsuhiko Maeda, Toshiyuki Kawakami, Hitoshi Nagatsuka

**Affiliations:** 1 Department of Oral Pathology and Medicine, Graduate School of Medicine, Dentistry and Pharmaceutical Sciences, Okayama University, Okayama 700-8558, Japan; gmd422094@s.okayama-u.ac.jp (K.T.); de18018@s.okayama-u.ac.jp (H.K.); de20052@s.okayama-u.ac.jp (S.Y.); jin@okayama-u.ac.jp (H.N.); 2 Department of Oral Pathology, School of Dentistry, Aichi Gakuin University, Nagoya 464-8650, Japan; hatsu@dpc.aichi-gakuin.ac.jp; 3 Hard Tissue Pathology Unit, Matsumoto Dental University Graduate School of Oral Medicine, Shiojiri 399-0781, Japan; toshiyuki.kawakami@mdu.ac.jp

**Keywords:** odontogenic tumor, pleomorphic adenoma, notch signaling, cell differentiation, immunohistochemistry, epithelial-mesenchymal interaction, malignant transformation

## Abstract

Histopathological findings of oral neoplasm cell differentiation and metaplasia suggest that tumor cells induce their own dedifferentiation and re-differentiation and may lead to the formation of tumor-specific histological features. Notch signaling is involved in the maintenance of tissue stem cell nature and regulation of differentiation and is responsible for the cytological regulation of cell fate, morphogenesis, and/or development. In our previous study, immunohistochemistry was used to examine Notch expression using cases of odontogenic tumors and pleomorphic adenoma as oral neoplasms. According to our results, Notch signaling was specifically associated with tumor cell differentiation and metaplastic cells of developmental tissues. Notch signaling was involved in the differentiation of the ductal epithelial cells of salivary gland tumors and ameloblast-like cells of odontogenic tumors. However, Notch signaling was also involved in squamous metaplasia, irrespective of the type of developmental tissue. In odontogenic tumors, Notch signaling was involved in epithelial–mesenchymal interactions and may be related to tumor development and tumorigenesis. This signaling may also be associated with the malignant transformation of ameloblastomas. Overall, Notch signaling appears to play a major role in the formation of the characteristic cellular composition and histological features of oral neoplasms, and this involvement has been reviewed here.

## 1. Introduction

Tooth germ is composed of epithelial and mesenchymal tissues, and epithelial–mesenchymal interactions are an essential process for tooth development. This complex process is associated not only with the development of teeth but also with the development of odontogenic neoplasms. Odontogenic tumors, like other tumors, are histologically classified into malignant and benign tumors. Malignant tumors include odontogenic carcinoma, odontogenic carcinosarcoma, and odontogenic sarcoma. Benign tumors include benign epithelial odontogenic tumors, benign mixed epithelial and mesenchymal odontogenic tumors, and benign mesenchymal odontogenic tumors.

Ameloblastoma is one of the most often encountered benign odontogenic epithelial tumors. It is clinically characterized as a benign but locally invasive tumor with a high recurrence risk. Histologically, it is characterized by a tumor nest similar to the enamel organ that constitutes tooth germ [1]. Ameloblastic fibroma is categorized as a benign mixed epithelial and mesenchymal odontogenic tumor composed of an ameloblastoma-like epithelial tissue and dental papilla-like mesenchyme, in which no dental hard tissue is present [2]. A calcifying odontogenic cyst is a developmental odontogenic cyst that was previously classified as an odontogenic mixed tumor. It is characterized by an epithelial lining of the cyst wall, which gives it a resemblance to the histological structure of the enamel organ that includes ghost cells and calcified foci within the odontogenic epithelial region. Dentin-like hard tissue and dysplastic dentin may be observed in the ectomesenchymal stromal tissue [3]. Odontogenic myxoma is a less common mesenchymal odontogenic tumor. Histopathologically, this tumor is characterized by a spindle or stellate cells proliferating in an abundant myxoid extracellular matrix with or without small masses of odontogenic epithelium [4].

Ameloblastic carcinoma is a rare malignant odontogenic tumor and is the malignant counterpart of ameloblastoma. This neoplasm combines a histological similarity to ameloblastoma with a strong cytological atypism [5].

Salivary gland tumors are common in oral neoplasms. Pleomorphic adenomas are especially prevalent in the neoplasm and arise from the salivary gland. They are classified as a benign epithelial salivary gland tumor [6]. Histopathologically, pleomorphic adenomas are characterized by an admixture of polygonal epithelial and neoplastic myoepithelial cells in a variable background stroma. Typically, a transition from the epithelial to the mesenchymal component creates characteristic myxomatous tissues around the glandular structure [6,7]. However, the neoplastic myoepithelial cells in tumor nests undergo further differentiation into plasmacytoid cells, squamous epithelioid cells, and chondrocytes, among other cell types. The characteristics of these oral neoplasms are shown in Table 1.

Notch is an evolutionarily-conserved pathway that plays a decisive role in cell fate determination in various tissue types during embryonic development, as well as postnatally [8]. Notch activity affects a wide range of developmental processes, such as differentiation, proliferation, apoptosis, neurogenesis, somite formation, hematopoiesis, angiogenesis, growth/differentiation of keratinocytes, and craniofacial development, including tooth development [9]. Under neoplastic conditions, such factors are also related to cell differentiation [10,11]. Therefore, immunohistochemistry analysis was performed based on the use of cell differentiation markers reported in previous papers. Muraki et al. [12] and Takamine et al. [13] performed immunohistochemistry analysis of ameloblastomas and pleomorphic adenomas to determine the role of Notch in cell differentiation. The results suggested that Notch is involved in cell differentiation in various tumors, partly leading to a histologically similar differentiation. Thus, in this review, we focused on the involvement of Notch in the cell differentiation of representative odontogenic tumors and pleomorphic adenomas using the literature previously published by our research group.

## 2. Histopathology of Ameloblastoma

Ameloblastoma is one of the most frequently encountered tumors arising from the odontogenic epithelium. It is characterized as a locally invasive tumor that is clinically benign but has a high risk of recurrence. Although the prognosis is good, a long-term follow-up survey is indispensable as the disease has been known to recur 10 years after the first treatment [1]. Histologically, ameloblastoma shows a follicular or plexiform pattern within a fibrous stromal connective tissue [1]. There are four basic histopathological variations: (1) conventional, (2) unicystic, (3) extraosseous/peripheral, and (4) metastasizing. There are two major conventional histological patterns: follicular and plexiform.

**Figure 1:**
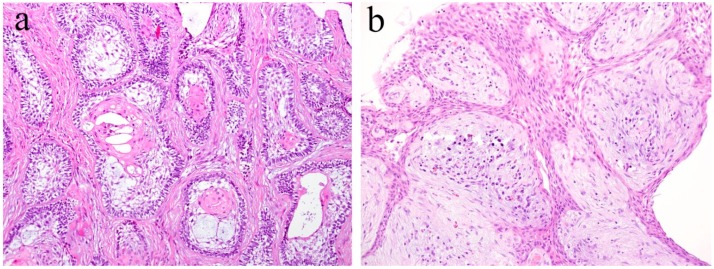
Histopathological features of ameloblastoma. The growth patterns of these tumors are classified as (**a**) follicular type and (**b**) plexiform type. HE staining, magnification ×40.

The follicular type ameloblastoma is the most common histological type, which is composed of island-like odontogenic epithelial tumor nests resembling the enamel organ of tooth germ. Epithelial tumor nests are surrounded by fibrous interstitial stromal tissue (Figure 1a). In the epithelial tumor nest of the follicular type ameloblastoma, tumor cells arranged at the periphery of these tumor nests are columnar, cuboidal, or hyperchromatic. Marginal cells are arranged in a palisade fashion to surround the tumor nest. The peripheral layered cells resemble the inner enamel epithelium. The cells located in the center of the tumor nest show a stellate reticulum-like structure. These structures exhibit a similarity to the enamel organ of tooth germ and result from the differentiation of tumor cells into tooth germ constituent cells. The central region of the tumor nest often undergoes squamous metaplasia, keratinization, and cystic changes. The tumor stroma consists of fibrous connective tissue. It is generally rich in collagenous fibers and has a low cell density.

The plexiform type ameloblastoma is the second-most common histological type and is composed of cord-like and anastomosing strands of odontogenic epithelial tumor nests. The tumor nests join with each other to form a network-like structure (Figure 1b). In the tumor nest of the plexiform type ameloblastoma, the cuboidal and/or columnar cells are arranged in the peripheral region of the anastomosing nests. The appearance of stellate reticulum cells are ambiguous in the center of the tumor nests. The tumor stroma consists of loose fibrous connective tissue. It generally shows an edematous and cyst-like stromal degeneration.

## 3. Notch Signaling in Ameloblastoma

**Figure 2:**
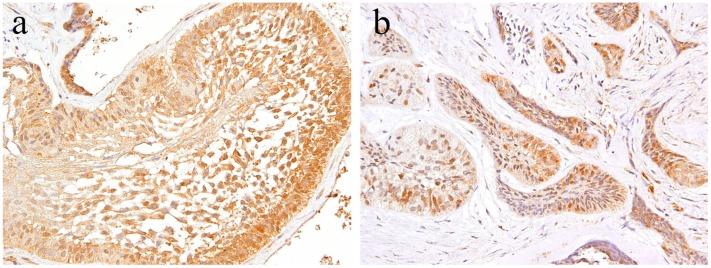
Immunohistochemical features of Notch1 in ameloblastoma. (**a**) Notch-positive reactions are observed in the cytoplasm of peripheral columnar cells and central squamous metaplastic cells. Magnification ×100. (**b**) Notch-positive reactions in the budding and peripheral layer of the epithelial nests. Magnification ×100. Anti-Notch-1 intracellular domain antibody, Abcam, ab83232, 1:100.

Notch plays an important role in cell fate determination in various tumors and also in normal tissue development. In the craniofacial area, the development of the mandibular condyles is an important feature; as such, we examined Notch-related factors in this region [14]. Under neoplastic conditions, Notch-related factors are also involved in cell differentiation [10,11]. Therefore, we also examined odontogenic neoplasms, such as benign ameloblastoma and ameloblastic carcinoma and its subtypes [12,15,16], squamous odontogenic tumor, odontogenic myxoma, ameloblastic fibroma [17], calcifying odontogenic cyst [18], and calcifying epithelial odontogenic tumors. In the above-mentioned studies, we histologically examined the expression of Notch and related factors and showed that it is involved in the development of odontogenic tumors and the differentiation of tumor cells as a potential role of Notch signaling. Our findings suggest that Notch1 plays a fundamental role in the cytological differentiation of epithelial and mesenchymal components and the acquisition of tumor-specific characters in these odontogenic tumors [12,15,16,18], but not in odontogenic myxoma [17]. In tooth development, the expression of Notch1 mRNA was detected in the odontogenic epithelium of mouse molar teeth, and was found to be transient in the inner enamel epithelium at the early bell stage and not in the differentiated ameloblast cells [9]. Notch1 and its ligand Jagged1 mRNA expression has been detected in human tooth germ at the early stage of dental crown calcification. Thus, Notch signaling has been shown to be involved in tooth development and odontoblast differentiation. In a previous study, ameloblastoma was found to express Notch1 and its ligand Jagged1 in polyhedral tumor cells in the central region of the tumor nest. The state of the Notch expression suggested that the tumor nest of ameloblastoma is similar to an early state of the enamel organ in tooth germ development. In a series of odontogenic tumor studies, we found that Notch was expressed in the squamous metaplasia of the odontogenic epithelium. Since Kumamoto and Ohki showed that the keratinizing cells were Notch negative, we believe the difference lies in the cell stages. In the squamous cell differentiation stage, the keratinizing cells showed the final stage of differentiation. On the contrary, squamous cells were found to be at the pre-final stage. In addition, our study detected Notch localization patterns in tumor cells. The expression of Notch1 in tumor tissue was localized to distinct parts of differentiation. In the periphery of the tumor nest of the ameloblastoma, Notch expression is high in the columnar or ameloblast-like cells and low in the cuboidal or basaloid cells. In the central region of the ameloblastoma cell nests, Notch expression and localization changes according to cell differentiation; however, Notch expression is low here compared to that in the marginal regions [12] (Figure 2). In particular, in ameloblastoma, Notch signaling is considered to play a major role in the differentiation of epithelial tumor cells. On the contrary, the expression of Notch is scarce in the stromal connective tissue of the tumor, suggesting that it does not play a positive role in tumor stroma formation.

## 4. Histopathology of Ameloblastic Fibroma and Odontogenic Myxoma

**Figure 3:**
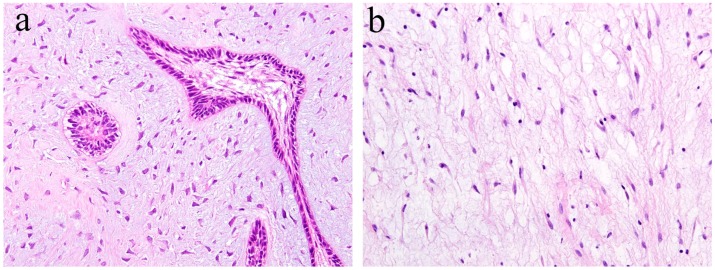
Histopathological features of (**a**) ameloblastic fibroma and (**b**) odontogenic myxoma. HE staining, magnification ×100.

Ameloblastic fibroma is a benign odontogenic tumor composed of odontogenic epithelial tissue and mesenchymal tissue. It is usually slow-growing, and the posterior area of the mandible is the most common location. It can cause the expansion of the jaw bone but rarely reaches a remarkable size. Histologically, the epithelial component shows a pattern of elongated strands of the epithelial tumor nest and the enamel organ-like tumor nest. The elongated strand tumor nest is composed of two tight and parallel-running layers of columnar and/or cuboidal cells. The thickening at the ends of strand tumor nests resemble the tooth bud. The cells surrounding the enamel organ-like tumor nest are columnar. The stellate reticulum is located in the central region of the tumor nest. The mesenchymal tumor component is cell-rich and myxoid and resembles the dental papilla of the tooth germ. In the mesenchymal region, spindle or stellate-shaped cells proliferate in a myxoid or mucoid extracellular matrix. These are frequently associated with an embedded tooth [2] (Figure 3a).

Odontogenic myxoma is the third-most frequent odontogenic tumor. It is classified as a benign mesenchymal odontogenic tumor and is most likely to occur in the molar region of the mandible. Clinically, it usually grows slowly and painlessly, and causes swelling of the jawbone. A recent retrospective case study reported that odontogenic myxoma occurring in the maxillofacial region tends to have a significant recurrence rate [4]. Radiographically, lesions appear as unilocular, multilocular, and scalloped radiolucencies, sometimes with root resorption. Histologically, the tumor tissue consists of randomly distributed stellate or spindle-shaped cells. Tumor cells produce an abundant alcianophilic extracellular matrix. These myxoid ground substances are rich in mucopolysaccharide and hyaluronic acid and contain small amounts of fine collagen fibers (Figure 3b).

The tumor cell component of odontogenic myxoma is simply derived from odontogenic ectodermal mesenchyme. The epithelial–mesenchymal interactions essential to tooth development are not involved in the development of odontogenic myxoma. On the contrary, during tumorigenesis, the ameloblastic fibroma is a tumor that is considered to have an epithelial–mesenchymal interaction between the epithelial component and the mesenchymal component of the tumor. The differentiation stage of the ameloblastic fibroma tumor is clearly different from the differentiation stage of the tumor, which lacks epithelial–mesenchymal interactions.

## 5. Notch Signaling in Ameloblastic Fibroma and Odontogenic Myxoma

**Figure 4:**
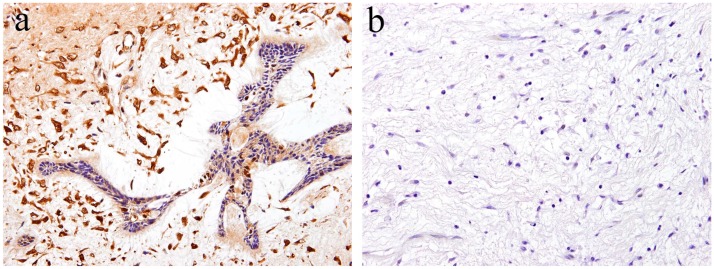
Immunohistochemical features of Notch1 expression. (**a**) Notch-positive reactions are observed in ameloblastic fibroma. Magnification ×100. (**b**) Notch expression is not observed in the tumor tissue of odontogenic myxoma. Magnification ×100. Anti-Notch-1 intracellular domain antibody, Abcam, ab83232, 1:100.

According to the literature [17], immunohistochemical analysis revealed that Notch expression is observed in both the epithelial and mesenchymal tumor tissue of ameloblastic fibroma. Within the epithelial tumor nests, a strong immunohistochemical Notch reaction is observed in the central region in comparison to the peripheral region. The immunohistochemical expression pattern of Notch in the epithelial component of the tumor is partially similar to that of ameloblastoma. However, the pattern of Notch expression in the columnar cells at the periphery of the tumor nest is different. Histologically comparing the follicular type of ameloblastoma with ameloblastic fibroma, there are a large number of ameloblast-like high columnar cells in the epithelial tumor nest of ameloblastic fibroma. These findings indicate that the differentiation stage of peripheral high columnar cells is more advanced in ameloblastic fibroma than ameloblastoma. In the follicular type of ameloblastoma, intense Notch expression appears in the peripheral columnar or cuboidal cells of the tumor nest. Moreover, in ameloblastic fibromas, Notch expression appears in the peripheral columnar cells; however, it is weak. Immunohistochemically, Notch signaling is believed to be involved in the differentiation of ameloblasts or columnar cells from the odontogenic epithelium. In the mesenchymal component of ameloblastic fibroma, a strong immunohistochemical positivity of Notch1 is observed in the tumor cells forming the dental papilla-like tissue (Figure 4a). The epithelial–mesenchymal interaction is an important event of cell proliferation and for differentiation during the development of various organs and tissues. During tooth development, epithelial–mesenchymal interactions are required, and Notch expression is observed in both odontogenic epithelial and mesenchymal tissues, suggesting its involvement in cell differentiation [19]. Our immunohistochemical examination revealed that Notch expression is not observed in the tumor tissue of odontogenic myxoma [17] (Figure 4b). These findings suggest that Notch signaling is involved in the epithelial–mesenchymal interaction of tumorigenesis of odontogenic tumors, and that the differentiation stage of the tumoral tissue in odontogenic myxoma is less advanced than that in ameloblastic fibroma.

## 6. Histopathology of Calcifying Odontogenic Cyst

**Figure 5:**
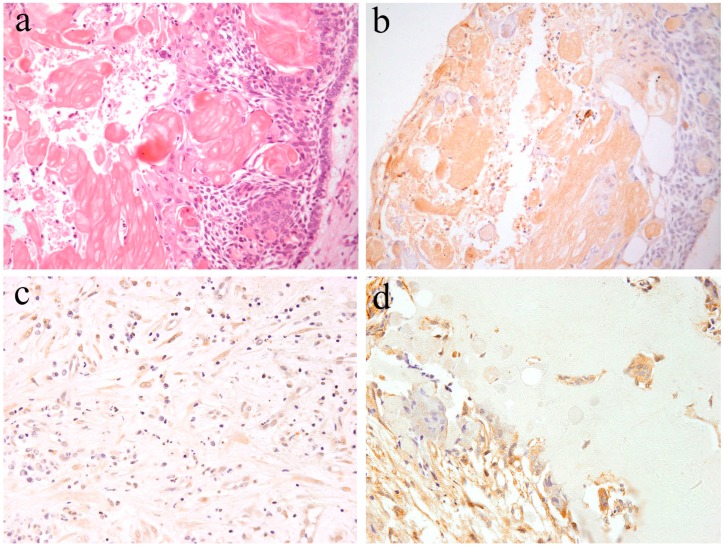
Microscopic features of (**a**) calcifying odontogenic cysts. HE staining, Magnification ×100. (**b**) Notch1-positive reactions are observed in both ghost cells and epithelial cells. Immunohistochemical staining, Magnification ×100. (**c**) Notch1-positive reactions are observed in ectomesenchymal spindle cells. Immunohistochemical staining, Magnification ×100. (**d**) Notch1-positive reactions are observed adjacent to dentinoid tissues. Immunohistochemical staining, Magnification ×100. Anti-Notch-1 intracellular domain antibody, Abcam, ab83232, 1:100.

Calcifying odontogenic cyst is a rare odontogenic cyst, and forms part of the group of ghost cell lesions of the jaw. Histologically, lesional tissues composed of a fibrous wall lined by ameloblastoma-like epithelium are characterized by cuboidal cells, basal cells, preameloblast-like cells, and stellate reticulum-like cells with scattered clusters of ghost cells and squamous metaplastic cells. Focal accumulations of ghost cells with calcifications may also be observed. The peripheral layered cells of the lined epithelial resemble the inner enamel epithelium. The cells located in the central region of the lined epithelial show a stellate reticulum-like structure. A variable amount of dentinoid may appears adjacent to the lining epithelium [3] (Figure 5). Occasionally, odontoma, ameloblastic fibroma, and adenomatoid odontogenic tumors can be detected in the same area.

## 7. Notch Signaling in Calcifying Odontogenic Cyst

Kawakami et al. reported histological Notch expression on calcifying odontogenic cysts [20,21]. Immunohistochemical analysis revealed that Notch expression is observed in the cytoplasm of both epithelial and mesenchymal cells. In the enamel organ-like epithelial component, Notch expression is similar to that of ameloblastoma. Epithelial cells, including ghost cells, express immunohistochemical Notch-positivity (Figure 5). Furthermore, the mesenchymal spindle and/or reticulum cells scattered in the stromal connective tissues express Notch-positivity (Figure 5). In the dentinoid formation area, Notch expression is present in the cytoplasm of adjacent cells of the dentinoid tissues and the embedded cells (Figure 5). According to the literature [20], Notch expression is also found in ghost cells. Notch localization has been previously found to be cytoplasmic and/or membranous, and Notch expression is also observed in metaplastic squamous cells. On the contrary, the adjacent epithelium expresses weak Notch positivity. These findings suggest that probably Notch signal activation in ghost cells exerts a lateral inhibitory effect on the adjacent epithelium, blocking them from adopting the same cell fate. In our study, Notch expression was not observed in any of the cases of odontogenic myxoma [17]. The loss of Notch expression suggests that neoplastic cells and tissues have no cytological differentiation. On the contrary, as in the case of calcifying odontogenic cysts, strong Notch expression has been detected in the odontogenic mixed tumor, ameloblastic fibroma. These Notch-related immunohistochemical positive features indicate that Notch signaling is closely involved in the epithelial–mesenchymal interaction and that cytodifferentiation occurs in calcifying odontogenic cysts.

## 8. Histopathology of Ameloblastic Carcinoma

**Figure 6:**
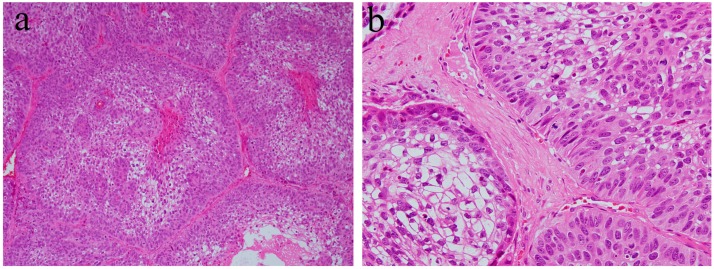
Histopathological features of ameloblastic carcinoma. (**a**) Follicular growth patterns of ameloblastic carcinoma. HE staining, magnification ×40. (**b**) Follicular tumor nests of ameloblastic carcinoma. HE staining, magnification ×100.

Ameloblastic carcinoma is a rare primary epithelial odontogenic malignant neoplasm. This neoplasm is characterized by a combination of the histological features of ameloblastoma and a strong cytological atypism. Ameloblastic carcinoma can have a follicular or plexiform pattern and can be formed of nests, sheets, or broad ribbons of tumor epithelium [5]. The histological characteristics are a tall columnar cellular morphology with pleomorphism, nuclear hyperchromatism, focal necrosis, and mitotic activity (Figure 6). The peripheral cell layer of the tumor nest shows palisading. Usually, reverse nuclear polarity is focally present. The organized stratification of columnar cells, stratum intermedium, and stellate reticulum found in ameloblastoma are lost (Figure 6). This histological tendency is more marked in higher-grade lesions. The central region of epithelial tumor nests or sheets may be replaced by an atypical basaloid epithelium or spindle epithelial cells. The central region of epithelial tumor nests may show acanthomatous metaplasia and cystic degeneration. In addition, ameloblastic carcinomas show a high proliferation index compared to ameloblastomas [5].

## 9. Notch Signaling in Ameloblastic Carcinoma

**Figure 7:**
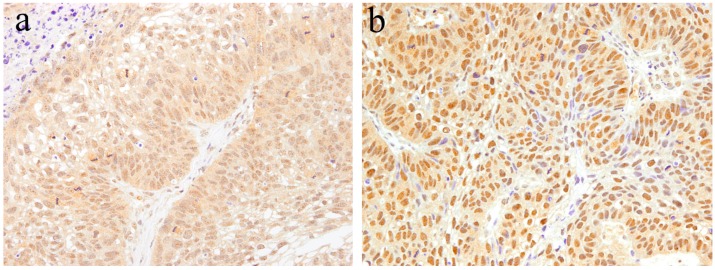
Immunohistochemical features of Notch1 expression in ameloblastic carcinoma. (**a**) Notch expression tends to be strong in the peripheral columnar cells. Positive reactions are mainly found in the cytoplasm. Magnification ×100. (**b**) Atypical cells, such as basaloid epithelium or spindle epithelial cells, show strong Notch expression. Positive reactions are mainly found in the nuclei. Magnification ×100. Anti-Notch-1 intracellular domain antibody, Abcam, ab83232, 1:100.

According to our immunohistochemical research [15], strong Notch-positive reactions are detected in most neoplastic cells of ameloblastic carcinoma. On the contrary, no Notch-positive reaction is found in the stroma. In the epithelial tumor nest, Notch expression tends to be strong in the peripheral columnar or cuboidal cells, and moderate in the central polyhedral cells (Figure 7). Contrarily, the central region of the epithelial tumor nests is replaced by atypical cells, such as basaloid epithelium, or spindle epithelial cells that show strong Notch expression (Figure 7). Immunohistochemically, there are two patterns in the cellular localization of Notch in ameloblastic carcinoma. One is a strong positive reaction in the cytoplasm of tumor cells (Figure 7), and the other is a strong positive reaction in the nuclei of tumor cells (Figure 7). In addition, the former has a higher cell proliferation activity and a larger number of mitotic figures, and the latter has a lower cell proliferation activity and a smaller number of mitotic figures. Ameloblastoma, which is the benign counterpart of ameloblastic carcinoma, usually does not appear in mitotic figures. Moreover, a few Ki-67 positive cells are observed in the proliferating tumor nests. In contrast, in the case of ameloblastic carcinoma, an increased number of Ki-67 positive cells have been found in almost all epithelial tumor nests. In certain cases of osteosarcoma, without epithelial components, Notch-positive reactions have been found in the neoplastic mesenchymal cells [15]. In our case study of osteosarcoma, the expression of Notch was found to be stronger in the well-differentiated regions compared to poorly differentiated regions [21]. These observations suggest that Notch signaling is apparent in cells when differentiation is in progress, and regulates cell differentiation and proliferation. Notch signaling is also associated with oncogenesis, whereby the impairment of its transmission leads to malignant transformation. The disturbance of the Notch signaling pathway has been detected in several cancers, such as breast cancer, esophageal cancer, and lymphoblastic acute leukemia (T-ALL) [22,23,24]. Furthermore, Notch has been the focus of studies on the metastasis, invasion, and malignant transformation of oral neoplasms, such as adenoid cystic carcinoma and malignant ameloblastoma [15,25]. Notch signaling is believed to be a major factor in the progression of malignant neoplasms.

In recent years, a cross-talk between the Wnt signal and the Notch signal has been reported [26]. Wnt signaling is an essential signal for tooth development, and ameloblastoma has also been found to express Wnt and its related factors [27]. In the process of Wnt signal transduction, the existence of a mechanism that stimulates Notch signaling has become clear. It has been reported that the Jagged1 ligand is upregulated downstream of Wnt signaling, indicating that a cross-talk between Wnt and Notch may be associated with tumor progression of ameloblastoma [28].

## 10. Histopathology of Pleomorphic Adenoma

**Figure 8:**
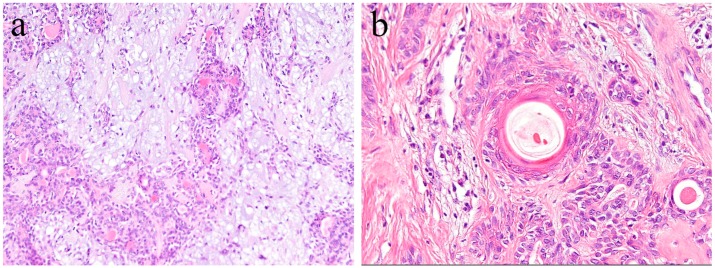
Histopathological features of pleomorphic adenoma (HE staining). (**a**) The tumor consists of solid sheet-like tumor nests and myxomatous mesenchymal parts. Magnification ×40. (**b**) Squamous metaplasia with keratin pearls located in the central region of the tumor nest. Magnification ×100.

In general, pleomorphic adenoma has a rounded shape and is covered with relatively thin fibrous connective tissues. Various types of cells, as well as a diverse range of differentiation, have been observed in this type of tumor. The tumor consists of small and/or large ductal-like/cystic spaces in the tissue. The majority of the tumor consists of glandular structures of tumor nests surrounded by fibrous tissue. Histologically, the tumor is separated from normal salivary gland tissue. Neoplastic parenchyma consists of duct-like and solid sheet-like structures composed mainly of tubular and myoepithelial cells. The inside of tumor nests is distinctively biphasic in appearance and consists of a sparse array of relatively loose cells and epithelial cell nests. Numerous cells are grouped together to form solid cell nests. Inside the tumor cell nest, a tube-like structure is observed. Bilayers of basal cells and large cubic cells form a tube-like structure (Figure 8). The mesenchymal area is a myxoma-like tissue and consists of myoepithelial cells, spindle shaped cells, and round or oval-shaped cells. Most of the cells of the mesenchymal region are derived from myoepithelial cells and dissociate from the tumor parenchyma to form myxoma-like tissues around the solid parenchymal region. In some areas, cartilage-like tissue is characteristically observed (Figure 8). In the myxomatous region, the myoepithelial cells sparsely form a cord-like structure, and the presence of mucus is confirmed. Myoepithelial cells differentiate into various tissues. Cartilage-like tissue is found in many cases of polymorphous adenomas. It has also been shown that this specialized tissue also originates from myoepithelial cells. In cartilage-like tissues, the presence of a specific cartilage matrix and cartilage-like cells is observed. The neoplastic epithelial cells dissociate from the mucus-like tissue via the secretion of their own substrate. Tumor parenchyma contributes to the formation of a myxomatous region. Tumor parenchymal cells coexist with, and are able to penetrate, the structures of mesenchymal cells and cartilage-like cells. Thus, epithelial components are dissociated and mixed into a mesenchymal-like tissue, resulting in a “mixed appearance.” In addition, tumor cells undergo significant squamous metaplasia.

## 11. Notch Signaling in Pleomorphic Adenoma

**Figure 9:**
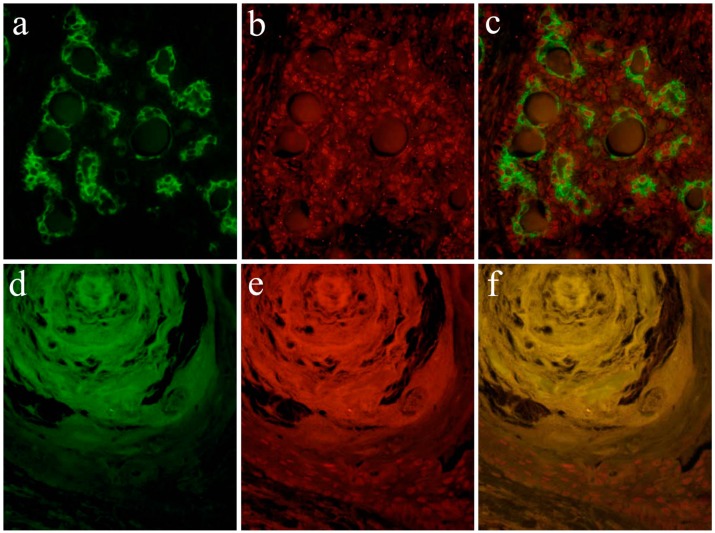
Immunofluorescent staining images of the formation of the ductal structure. (**a**) Green color indicates the localization of CK7, and is found in ductal cells. (**b**) Red color indicates the Notch1 localization, and (**c**) merged image of CK7 and Notch1.; Immunofluorescent staining images of squamous metaplasia. (**d**) Green color indicates the CK13 localization, and is found in squamous epithelial cells. (**e**) The nuclear localization of Notch1 is shown in red color in basal cells, and (**f**) merged image of CK13 and Notch1. Magnification ×100. Anti-Cytokeratin 7 antibody, abcam, [RCK105], ab9021, 1:100; anti-Cytokeratin 13 antibody, abcam, [AE8], ab16112, 1:100; anti-Notch-1 intracellular domain antibody, Abcam, ab83232, 1:100.

According to the literature [13], Notch expression is found in the cytoplasm and nuclei of ductal epithelial cells and in neoplastic myoepithelial cells. Keratins are useful epithelial differentiation markers due to their expression being cell type-specific and differentiation-specific. Cytokeratin 7 (CK7) is known to be expressed in ductal epithelial cells. In the pleomorphic adenoma, neoplastic ductal epithelial cells expressing CK7 and Notch are also expressed in the nuclei of several ductal epithelial cells. Double immunofluorescent staining revealed that Notch and CK7 are co-expressed in neoplastic ductal structures. Notch is expressed in the majority of the nucleus of neoplastic cells in the solid lesions of the tumor nest. Cytokeratin 13 (CK13) is normally expressed in squamous epithelial cells. In tumor cells that have formed squamous metaplasia, CK13 showed a strong expression in the squamous cell layer. At the site of squamous metaplasia, histological differentiation from basal cells to squamous epithelium was observed. Notch is strongly expressed in the cytoplasm and nucleus of basal cells, and its expression is weakened as squamous epithelial cells differentiate. As the result of double immunofluorescent staining of Notch and CK13, CK13 was found to be negative in basal cells at the periphery of the squamous metaplastic region but nuclear Notch was prominently positively expressed, where CK13-positive squamous cell lost their nuclear positive expression of Notch (Figure 9).

Notch signaling is a major signaling pathway that controls cell fate and tissue growth. Notch is a single pass transmembrane receptor with domains outside and inside the cell membrane. When Notch binds to its ligand, the Notch intracellular domain (NICD) is cleaved and moves into the nucleus. This conveys instructions to various morphogenesis and tissue differentiation processes during development. Notch plays an important role in intercellular signaling and is involved in the maintenance, differentiation, and neural function of stem cells in adults. Disturbances in the Notch signaling pathway are associated with oncogenesis, including esophageal cancer and breast cancer, among others [10,22]. Notch is also involved in the metastasis of malignant tumors, such as adenoid cystic carcinoma and malignant ameloblastoma [15,25], and is considered to be a major factor in the progression of malignant neoplasms. On the contrary, it has been suggested that Notch-Jagged1 is the primary mechanism for determining the fate of ghost cells, one being special keratinization [20]. Keratins are structural proteins that make up the cytoskeleton of keratinocytes, and Notch may be involved in the expression of cytokeratin. The expression of CK7 and Notch in ductal epithelial cells suggests that Notch is involved in the neoplastic ductal cell differentiation of pleomorphic adenoma [29]. The expression of Notch was strong in basal cells, and was attenuated during their differentiation to squamous cells. This change in expression suggests that Notch is involved in the differentiation of basal cells into squamous cells [30,31]. In addition, the loss of expression of keratinocytes in the nucleus denotes the end of cell differentiation. In Okuda et al.’s study, the expression of Wnt was confirmed in small basal cells forming the ductal epithelium, with basal cells producing squamous epithelialization. This result is consistent with our current study, suggesting that Notch functions at sites where cell differentiation takes place [32,33]. Our data on the expression of Wnt in 30 cases of pleomorphic adenomas showed that Wnt is expressed in the ductal structure and squamous metaplasia of solid tumor nests. In these regions, the β-catenin pathway was activated, which strongly suggested that Wnt is involved in squamous metaplasia and differentiation of duct-like structures [32]. Notch signaling could, therefore, undergo cross-talk with Wnt signaling to play an important role in the cell differentiation of various tumors [34,35].

## 12. Conclusions

**Figure 10:**
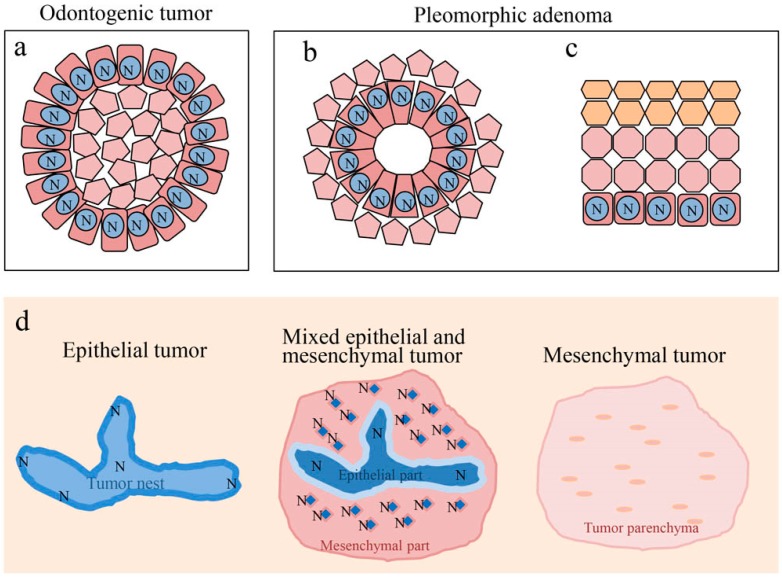
Notch signaling in cell differentiation of oral tumors. (**a**) Differentiation of ameloblast-like cell. (**b**) Differentiation of ductal epithelium. (**c**) Differentiation of squamous epithelium. (**d**) Notch signaling is involved in epithelial-mesenchymal interactions of odontogenic tumors. N: Notch expression.

Ameloblastoma, ameloblastic fibroma, calcifying odontogenic cyst, odontogenic myxoma, ameloblastic carcinoma, and pleomorphic adenoma show disease-specific histological features. Although these histological differences are caused by a variety of factors, Notch is considered to play a central role. In odontogenic tumors and cysts, Notch signaling is believed to be involved in the cytological differentiation of ameloblast-like columnar cells. In pleomorphic adenomas, Notch signaling is believed to be involved in the cytological differentiation of neoplastic cells that form duct-like structures. Moreover, Notch is involved in the differentiation of squamous metaplasia. In all of the tumors studied, Notch was found to be strongly expressed in cells undergoing differentiation, and weakly expressed in cells that had already differentiated. Furthermore, the findings discussed here suggest that Notch signaling induces the differentiation of odontogenic cells through epithelial-mesenchymal interactions (Figure 10). Notch signaling is also associated with oncogenesis and the impairment of its signaling may be associated with the malignant transformation of ameloblastic carcinoma. In the pleomorphic adenoma, the Wnt expression results coincided with the function of Notch at the sites of cell differentiation. Regarding cell differentiation resulting from Wnt signaling, Notch signaling may undergo a cross-talk with Wnt signaling to play an important role in tumor cell differentiation. This cross-talk between Wnt and Notch may be associated with the tumor progression of ameloblastoma.

## Figures and Tables

**Table 1:** Clinical and histological features in different type of oral neoplasms.

	Clinical Features	Histological Features
Odontogenic tumor/cyst		
Ameloblastoma	Locally invasive tumor. Clinically benign but has a high risk of recurrence.	Epithelial tumor nests are similar to the enamel organ. Tumor cells arranged at periphery of tumor nests are columnar or cuboidal.
Ameloblastic fibroma	Usually grows slowly and painlessly, causing swelling of the jawbone.	Tumor composed of ameloblastoma like epithelial tissue and dental papilla like mesenchyme.
Odonotogenic myxoma	Usually grows slowly and painlessly, causing swelling of the jawbone. Clinically benign but has a high risk of recurrence.	Spindle or stellate tumor cells proliferating in an abundant myxoid extracellular matrix.
Calcifying odontogenic cyst	Usually grows slowly and painlessly, causing swelling of the jawbone.	Ameloblastoma-like lining epithelium including ghost cells and calcified foci. Dentin-like hard tissue and dysplastic dentin may be observed.
Ameloblastic carcinoa	Rare odontogenic carcinoma. Most cases arise in posterior segments of the jaws.	Histological similarity of ameloblastoma with strong cytological atypism.
Salivary gland tumour		
Pleomorphic adenoma	Tumor shows slowly and painless growing. Most cases arise in the parotid.	Admixture of polygonal epithelial and neoplastic myoepithelial cells in a variable background stroma.
